# Patient-Centered Decision Support: Formative Usability Evaluation of Integrated Clinical Decision Support With a Patient Decision Aid for Minor Head Injury in the Emergency Department

**DOI:** 10.2196/jmir.7846

**Published:** 2017-05-19

**Authors:** Edward R Melnick, Erik P Hess, George Guo, Maggie Breslin, Kevin Lopez, Anthony J Pavlo, Fuad Abujarad, Seth M Powsner, Lori A Post

**Affiliations:** ^1^ Department of Emergency Medicine Yale School of Medicine New Haven, CT United States; ^2^ Department of Emergency Medicine Mayo Clinic Rochester, MN United States; ^3^ Yale School of Medicine New Haven, CT United States; ^4^ School of Visual Arts New York, NY United States; ^5^ Department of Psychiatry Yale School of Medicine New Haven, CT United States; ^6^ Department of Emergency Medicine Feinberg School of Medicine Chicago, IL United States

**Keywords:** clinical decision support, decision aids, head injury, minor, medical informatics, spiral computed tomography, health services overuse, patient-centered outcomes research

## Abstract

**Background:**

The Canadian Computed Tomography (CT) Head Rule, a clinical decision rule designed to safely reduce imaging in minor head injury, has been rigorously validated and implemented, and yet expected decreases in CT were unsuccessful. Recent work has identified empathic care as a key component in decreasing CT overuse. Health information technology can hinder the clinician-patient relationship. Patient-centered decision tools to support the clinician-patient relationship are needed to promote evidence-based decisions.

**Objective:**

Our objective is to formatively evaluate an electronic tool that not only helps clinicians at the bedside to determine the need for CT use based on the Canadian CT Head Rule but also promotes evidence-based conversations between patients and clinicians regarding patient-specific risk and patients’ specific concerns.

**Methods:**

User-centered design with practice-based and participatory decision aid development was used to design, develop, and evaluate patient-centered decision support regarding CT use in minor head injury in the emergency department. User experience and user interface (UX/UI) development involved successive iterations with incremental refinement in 4 phases: (1) initial prototype development, (2) usability assessment, (3) field testing, and (4) beta testing. This qualitative approach involved input from patients, emergency care clinicians, health services researchers, designers, and clinical informaticists at every stage.

**Results:**

The *Concussion or Brain Bleed* app is the product of 16 successive iterative revisions in accordance with UX/UI industry design standards. This useful and usable final product integrates clinical decision support with a patient decision aid. It promotes shared use by emergency clinicians and patients at the point of care within the emergency department context. This tablet computer app facilitates evidence-based conversations regarding CT in minor head injury. It is adaptable to individual clinician practice styles. The resultant tool includes a patient injury evaluator based on the Canadian CT Head Rule and provides patient specific risks using pictographs with natural frequencies and cues for discussion about patient concerns.

**Conclusions:**

This tool was designed to align evidence-based practices about CT in minor head injury patients. It establishes trust, empowers active participation, and addresses patient concerns and uncertainty about their condition. We hypothesize that, when implemented, the *Concussion or Brain Bleed* app will support—not hinder—the clinician-patient relationship, safely reduce CT use, and improve the patient experience of care.

## Introduction

After a patient sustains a minor head injury, computed tomography (CT) imaging can diagnose structural brain injuries like hemorrhages but cannot detect the presence or severity of concussion [1]. The Canadian CT Head Rule, a clinical decision rule designed to safely reduce imaging in minor head injury, was developed and validated but has not decreased CT use [2-4]. This rule is 100% sensitive for predicting the need for neurosurgical intervention and more specific than other guidelines [3,5-7]. It should decrease CT use by one-third, making care more affordable, efficient, and safer [8-12]. The American Board of Internal Medicine and the American College of Emergency Physicians Choosing Wisely Initiative recommends avoiding unnecessary head CTs in emergency department (ED) patients with minor head injuries as the top national priority for addressing overuse in emergency care [13]. Conversely, CT imaging rates increased, and clinical decision support (CDS) implementation efforts have only had a modest effect (5%-8%) on decreasing CT use [4,14,15]. Research on nonclinical factors that influence overuse of CT revealed clinicians and patients identified establishing trust, patient engagement, and reassurance as essential to decreasing overuse of imaging [16,17].

Empathic care requires tools that facilitate conversation between patient and clinician [17-19]. Unfortunately, contemporary electronic health records (EHRs) tend to impede conversation [20-24]. The EHR interface physically separates the clinician from the patient, compromising communication. It distracts and decreases eye contact, touch, and decreases patient time with clinicians [20-23] and focuses almost entirely on physician behavior even if it is patient-specific (and evidence-based). Informing patients directly has rarely been part of the effort [25-27]. CDS is most effective when it is part of the clinician workflow at the time and location of decision making [27,28]. Patient decision aids, on the other hand, focus on patients, trying to help them decide among options by clarifying patient values, preferences, and goals and providing the best scientific evidence available to increase understanding of possible risks, benefits, alternatives, and their associated outcomes [29]. A successful decision aid facilitates conversation between the patient and clinician and improves patient engagement [18].

Current EHRs prohibit empathic care. Technology must support—not hinder—the clinician-patient relationship. Although paper charts were intuitive and simple, they were criticized for being disorganized and illegible, leading to medical errors. EHRs promised to improve patient safety and outcomes by reducing errors. In the rush to adopt EHRs to qualify for federal incentive payments, clinicians and hospitals adopted products with poor usability and poor integration that impede clinical workflow [30]. The EHR's potential for improving care has not yet been realized [27,28]. A large-scale study of EHR implementation found no negative association with mortality or adverse events across 17 hospitals [31]. EHR implementation has done harm in other ways [21,24,30]. Ratanawongsa et al [21] found high computer use by clinicians to be associated with lower patient satisfaction and communication. Sinsky et al [22] also found that physicians only spend 27% of their time face to face with patients, with 49% of their time spent on the EHR and desk work. In addition, EHR documentation requires an additional 1 to 2 hours daily of after-hour charting. A productivity analysis in the emergency care setting found that data entry accounted for 43% of physician time, requiring 4000 mouse clicks per shift [23]. Furthermore, EHRs in their current form physically obstruct and separate the clinician and patient, denying patients time with their clinician as well as compromising communication and human connection by distracting and decreasing eye contact and touch [20-23]. We propose that the patient-centered decision support presented here is the first step toward a more empathic medical interface that can support the clinician-patient relationship.

We developed a computerized, user-centered decision support tool called *Concussion or Brain Bleed* [32] for use on tablet computers (with 1536 × 2048 resolution) that integrates a patient decision aid and CDS at the bedside for decisions about CT use in ED patients with minor head injury. Herein is the design, development, and user experience and user interface (UX/UI) evaluation of *Concussion or Brain Bleed*. *Concussion or Brain Bleed* aims to engage patients in their care by giving them an understanding of their condition and helping them trust their clinician to safely reduce CT use in minor head injury.

## Methods

### Design

A user-centered design approach based on UX/UI industry standards was followed to develop a decision tool to promote shared decision making [33-35]. User-centered design is an iterative, multistage design and evaluation approach that is driven and refined by user input and customizes the interface based upon an explicit understanding of users, tasks, and environments [36]. UX design refers to user experience, while UI design stands for user interface. Both elements are crucial to app development. UX/UI refers to different aspects of the design. UX design is more comprehensive than UI—encompassing user needs, values, abilities, and limitations as they relate to the user’s interaction with and perception of the design product. UI design focuses on ensuring that the graphical interface has elements that can be used, accessed, and understood based on user needs. UX/UI development was adopted with a goal of creating a tool that deviates from traditional CDS (eg, alerts and reminders).

**Figure 1:**
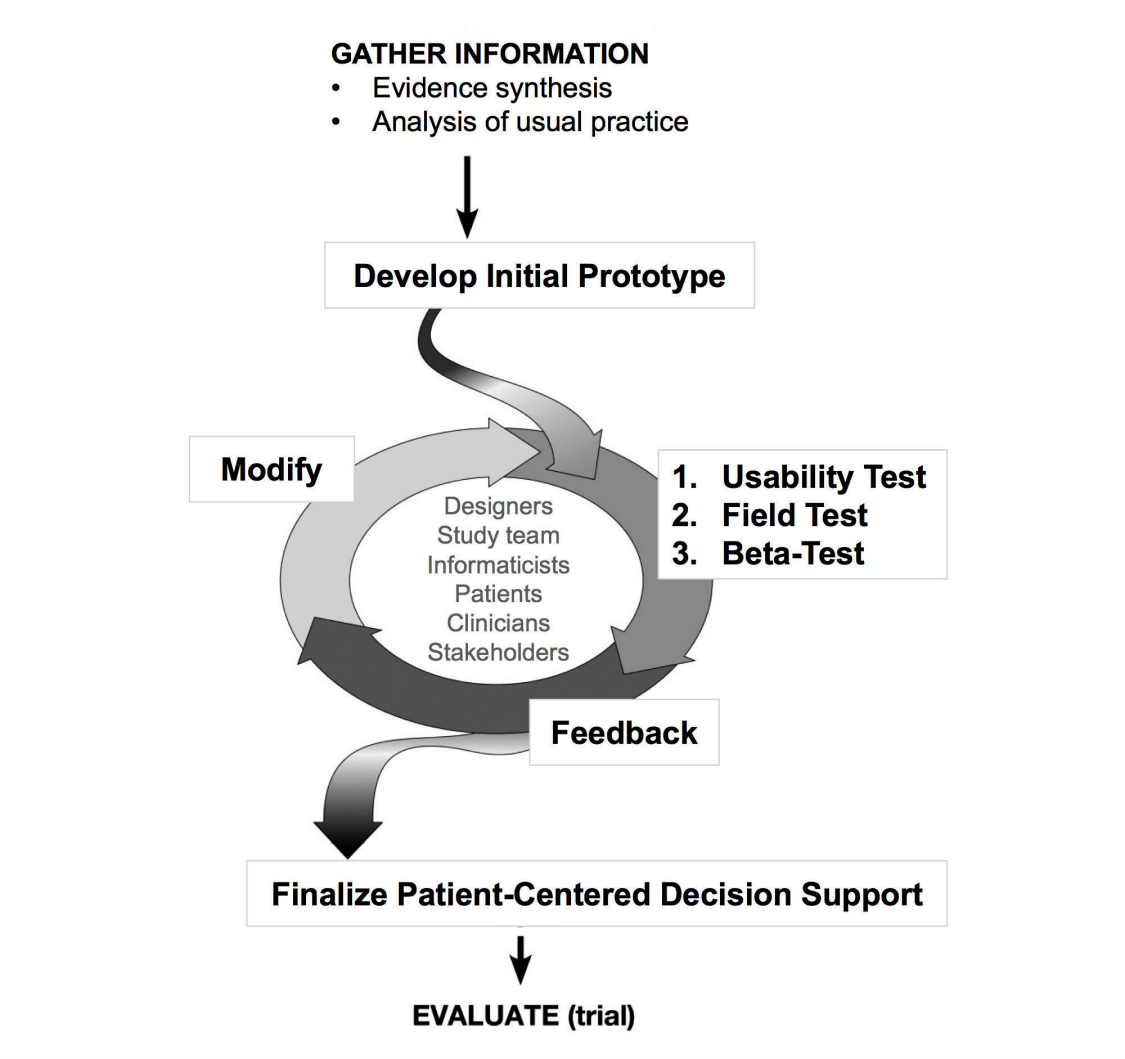
Patient-centered decision support development process.

UX/UI design elicits feedback and input from a multidisciplinary team, here including patients, emergency care clinicians (attending physicians, residents, physician assistants, and nurse practitioners), health services researchers, interaction design experts, and health systems information specialists (including a software system engineer and a computer programmer) to make incremental refinements to the prototypes. The development process involved successive iterations of the prototype within 4 UX/UI phases: (1) initial prototype development, (2) usability testing, (3) field testing, and (4) beta testing (Figure 1). Each phase continued until thematic saturation [37]. The initial prototype, including review and synthesis of the evidence and analysis of usual practice, has been previously described [16,19]. In the second phase, we performed formative usability evaluation in a simulated clinical environment using clinicians with standardized patients to maximize ease of use and clinical integration. Next, the prototype was field-tested by the research team with ED patients and, finally, the tool was beta-tested during clinical care by physician users.

### Study Setting and Population

Participants were patients and clinicians recruited from an urban, academic Level I trauma center ED with 103,000 patient visits per year and a satellite ED with 24,000 patient visits per year. Clinicians were recruited from the 48 attending physician faculty, 58 resident physicians, and 47 midlevel providers.

### Protocol

The study protocol was approved by the hospital institutional review board (IRB). All participants provided their verbal consent as specified by the IRB. Some portions of the evaluation were performed at an outside institution. The protocol was also approved by that institution’s IRB. Usability evaluation subjects were compensated for their time and travel with $100 gift cards. In beta testing, physicians were compensated for their time with $50 gift cards for each patient enrolled. Patients were not compensated in beta testing during their ED visit.

### User-Centered Design

#### Development of Initial Prototype

The full details of the initial development process including review and synthesis of the evidence and analysis of usual practice are reported elsewhere [16,19]. To identify nonclinical human factors that promote or inhibit appropriate use of CT in patients presenting to the ED with minor head injury, we performed qualitative studies in 3 phases: (1) patient focus groups, (2) clinician focus groups, and, (3) cognitive task analysis with direct ED observation and individual semistructured interviews using the critical decision method [16]. Next, a multidisciplinary team applied the findings from the qualitative study as user requirements for the initial prototype [19]. Primary goals were to promote smooth navigation through screens while completing tasks of patient education, risk communication, and shared decision making in the ED.

#### Usability Evaluation

Formative usability evaluations were conducted in a simulated environment to observe, record, and analyze a standardized clinician-patient encounter with the prototype. Using a “think aloud” protocol, scripted simulations of patient encounters with clinicians and standardized patients were observed and analyzed [38]. Attending emergency physicians were given a case study (Multimedia Appendix 1) to use the prototype while commenting on what they saw, thought, did, and felt. Inferences were made about the reasoning process behind task completion. Afterwards, a usability feedback questionnaire and semistructured interview (Multimedia Appendix 2) were conducted to determine the tool’s ease-of-use, usefulness, and how the decision-making process was affected by the tool.

#### Field Testing

To optimize naturalistic decision making under the constraints of the complex, high-pressure ED, field testing was conducted by the research team. ED patients available and amenable to participation were identified by the treating clinicians on duty. The prototype was implemented and reviewed by patients during their clinical encounter when they were not actively under evaluation. Patterns of conversation were analyzed while issues and challenges with the tool’s use were noted; all notes and experiences were shared and used to track the performance of successive iterations of the prototype based on content and quality of the conversation between the study clinician and the patient. Patients completed a semistructured interview (Multimedia Appendix 3) regarding the tool’s content and format within the ED context. The tool was iteratively refined according to ecological interface design to optimize communication of patient-specific risk [39,40]. After thematic saturation, the wireframe prototype was programmed for use as a Web app on an iPad (Apple Inc). Technical specifications and system requirements were similar to the initial prototype [19].

#### Beta Testing

Beta testing was conducted by emergency physicians using the interactive prototype during clinical care of ED patients with minor head injury. Physicians described their experience to improve workflow. Structured email interviews were conducted after physicians had seen multiple patients. Survey responses informed the final prototype.

## Results

*Concussion or Brain Bleed* underwent 16 successive revisions with content, process, and format adjustment based on usability, field, and beta testing.

### Development of Initial Prototype

The initial results of the prototype were previously reported [16,19]. Cognitive task analysis (critical decision method interviews and 150 hours of direct observation in the ED of peer-nominated senior emergency physicians recognized for their skill in safely minimizing testing while maintaining patient safety and engagement) revealed 5 core domains: trust, anxiety, constraints, influence of others, and patient expectations [16].

**Figure 2:**
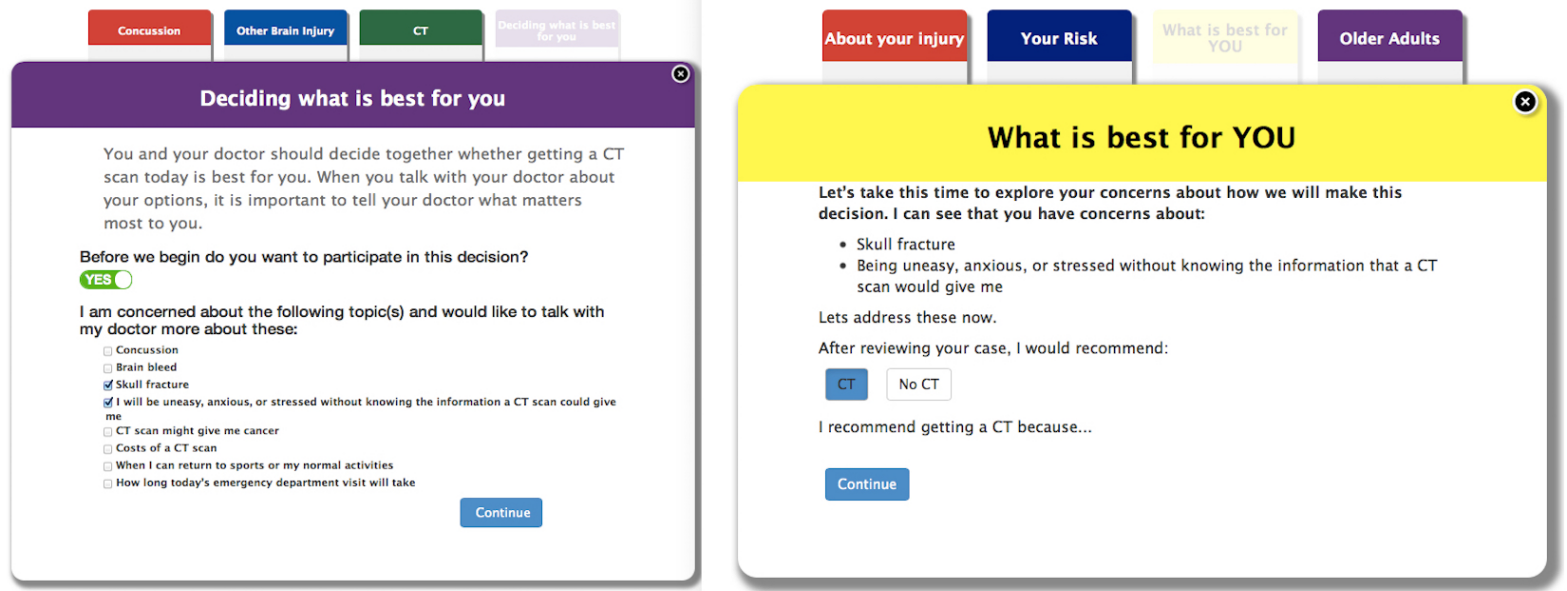
Initial prototype screen capture of patient concern screens.

The initial prototype followed a visual metaphor of design reminiscent of decision aids on paper cards [19]. After the patient filled out eligibility and questionnaire forms to autopopulate subjective components of the clinical decision rule, 3 sections followed. The first section centered around patient education (information about concussions, CT scans) to be used by the patient alone prior to the clinician’s evaluation and gave the patient the opportunity to flag concerns on a digital checklist. These concerns would later show up in the second section to be used by the clinician with the patient (screen capture of this section displayed in Figure 2). After completing a CDS checklist, the tool generated patient-specific risk estimates for pertinent outcomes and risk of cancer from a head CT. The final section involved a process of shared decision making in which patients and clinicians decided together whether to obtain a CT scan, to continue to be observed in the ED, or to go home.

### Usability Evaluation

Usability evaluation was conducted 3 times with 9 users. Observation revealed the tool required modification to facilitate conversation between the patient and clinician to be incorporated seamlessly into the clinical workflow [18,19]. Therefore, the initial user-centered design was augmented by interaction design using patient-centered and participatory decision aid development [18,19,41-44]. An interaction designer (MB) joined the research team [18,45,46]. Subsequent rounds involved rapid prototyping and low-fidelity wireframing.

**Figure 3:**
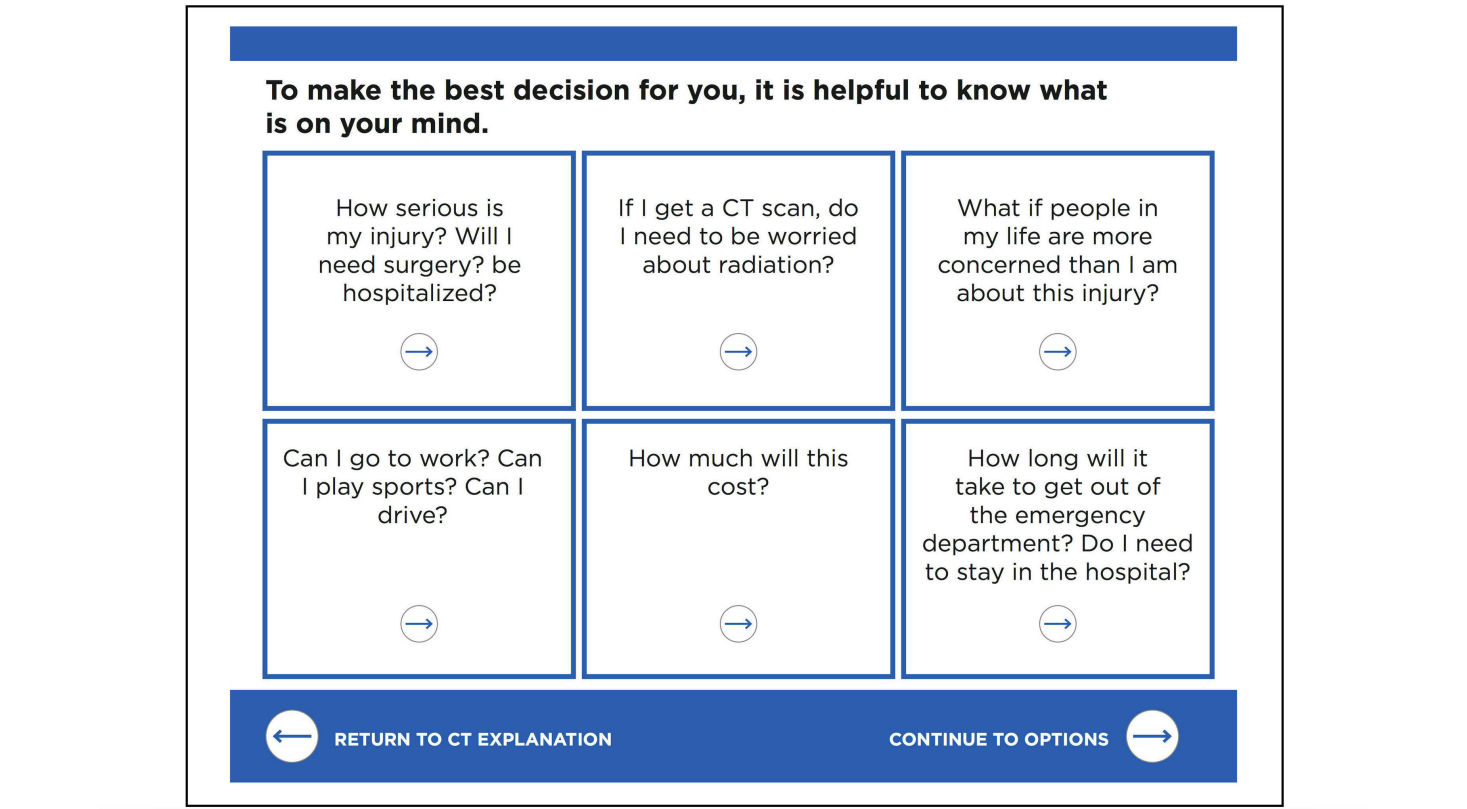
Revised Concerns section after initial round of usability evaluation.

This enhanced approach focused on tool usefulness (and lack of use by test subjects). Interview responses revealed users were not using the tool because the tool was overly prescriptive with too much text on the screen that interrupted or distracted from conversation with patients. Earlier prototypes were overdesigned, which forced clinicians to give more attention to the tool than the patient or to abandon the tool. Eliminations included the patient section with educational materials for patient review prior to the clinician’s evaluation (based on previous qualitative findings that patients come to the ED for a clinician’s expert evaluation) and a patient demographic survey and questionnaire about the injury. Revisions dramatically reduced the number of screen taps, checkboxes, and data entry. Furthermore, the Concerns section expanded to 6 boxes a patient could select to discuss (Figure 3). This minimalist version allowed clinicians to adapt the tool to their practice style and patient-specific education. It reassured patients by providing structure to the clinical conversation with cues (eg, How soon can I get back to work?). The tool was less prescriptive and increased the likelihood of implementation.

### Field Testing

**Figure 4:**
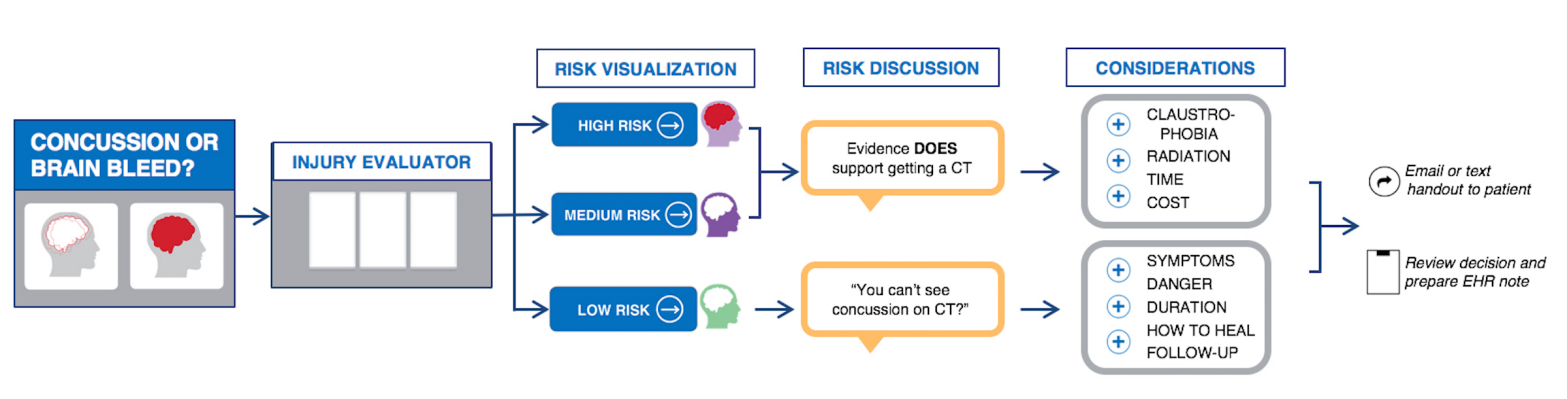
Conceptualization of the workflow and potential pathways for the Concussion or Brain Bleed application.

Field testing was conducted with 10 patients. Additional incremental revisions were made to the prototype. Observation and analysis of use in the ED context and application of ecological interface design principles distilled the workflow for the final *Concussion or Brain Bleed* app (Figure 4). This further elucidated important patient issues. The final app now supports the clinician’s decision and patient engagement and education around patient-specific risk about head injuries, CT imaging, counseling, and patient concerns.

**Figure 5:**
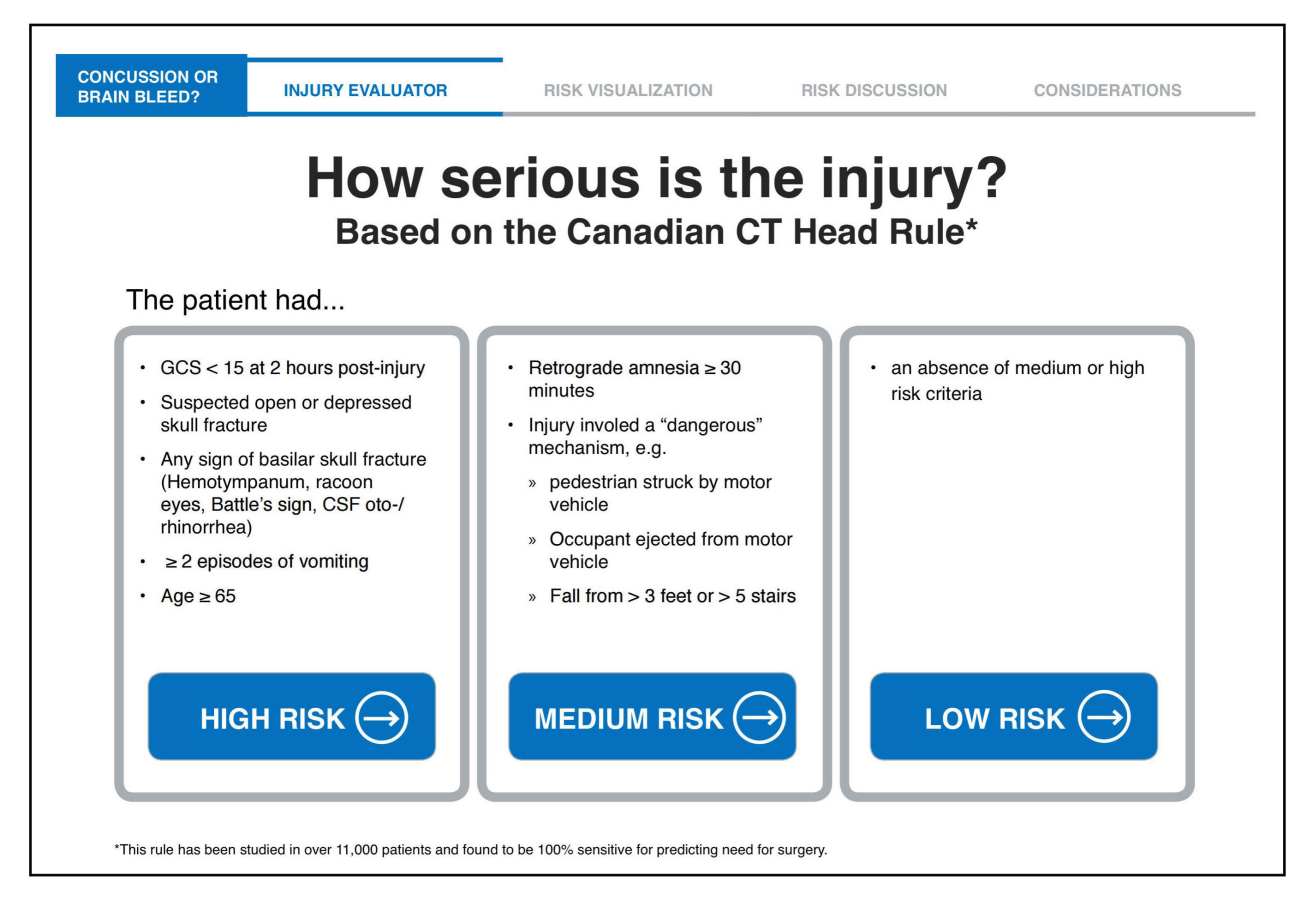
Clinical Decision Support portion of app after field testing.

Data entry was streamlined, and explicit user input was nearly eliminated. Grouping risk categories provides the clinician with the patient’s individualized risk assessment by a single tap of the screen (Figure 5). This efficient Canadian CT Head Rule display gives the clinician more time for risk communication with the patient.

**Figure 6:**
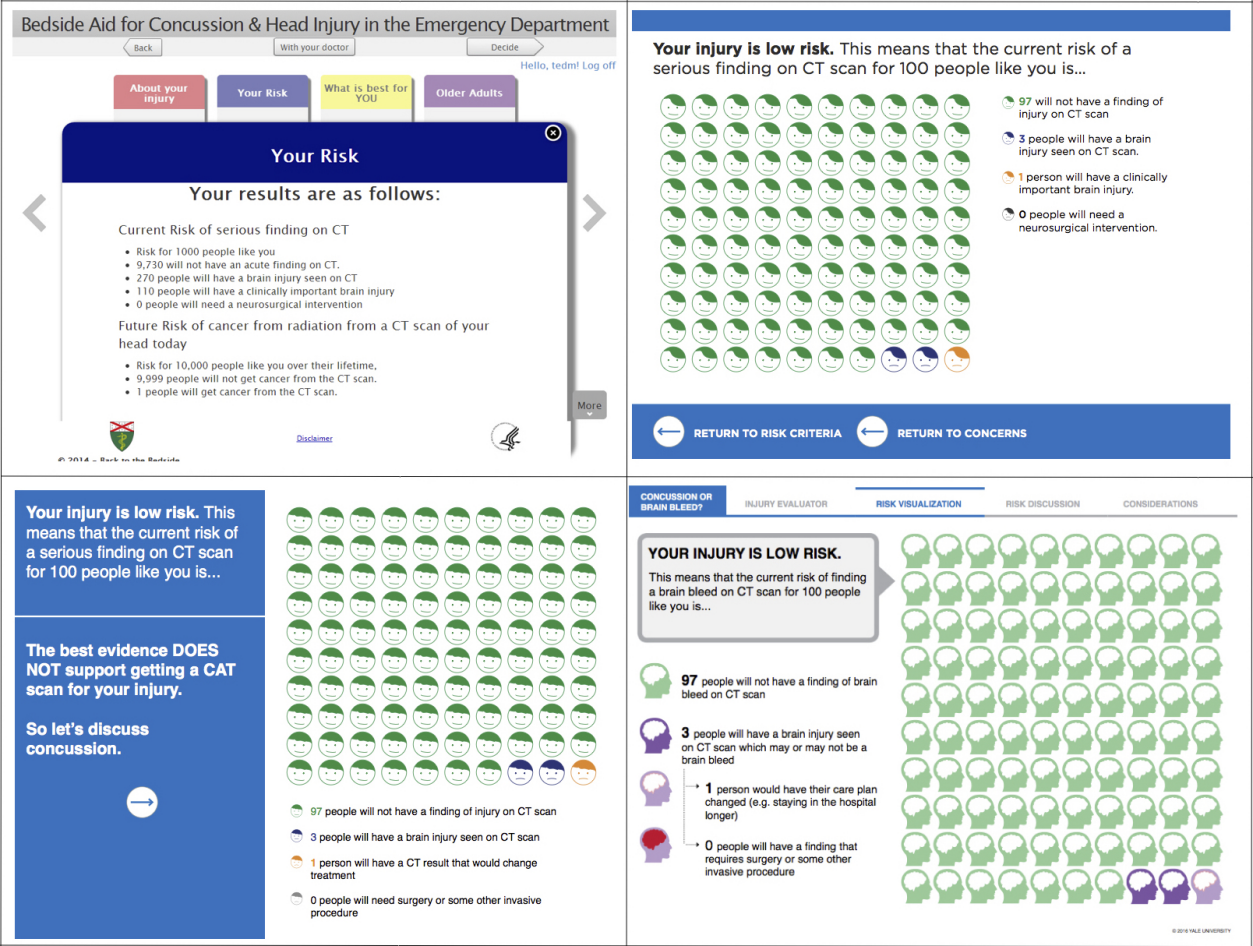
Risk visualization for low-risk patients from the initial prototype (top left) through usability testing (early, top right; late, bottom left) and field testing (bottom right).

The risk visualization format and content underwent revisions from the initial prototype through usability and field testing (Figure 6 a-d). The initial prototype used text-based risks (eg, clinically important brain injury) [2,19,47]. Later versions used pictographs, plain language, absolute risks with a constant denominator, and a color scheme to differentiate the 4 categories of patient-centered outcomes [42,44,48,49].

**Figure 7:**
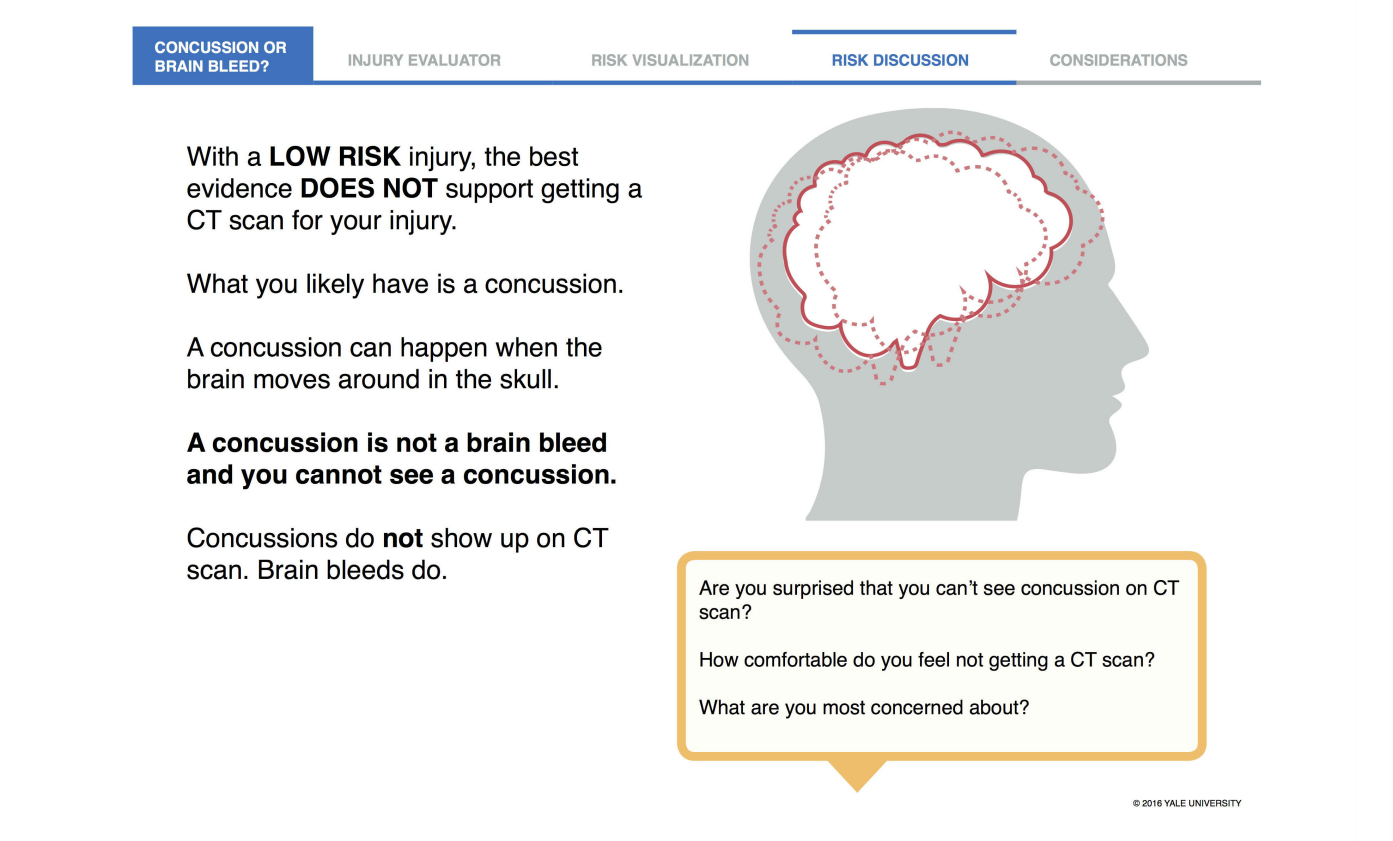
Risk discussion screen for low-risk patients after field testing.

A key finding was how important it is to teach and emphasize that a concussion is not visible on CT. The tool evolved into helping patients understand specific recommendations and their implications. The Risk Discussion section offers plain language on the utility (or lack thereof in low-risk patients) of CT as well as cues to discuss concussion and the individual patient’s concerns (Figure 7).

### Beta Testing

**Figure 8:**
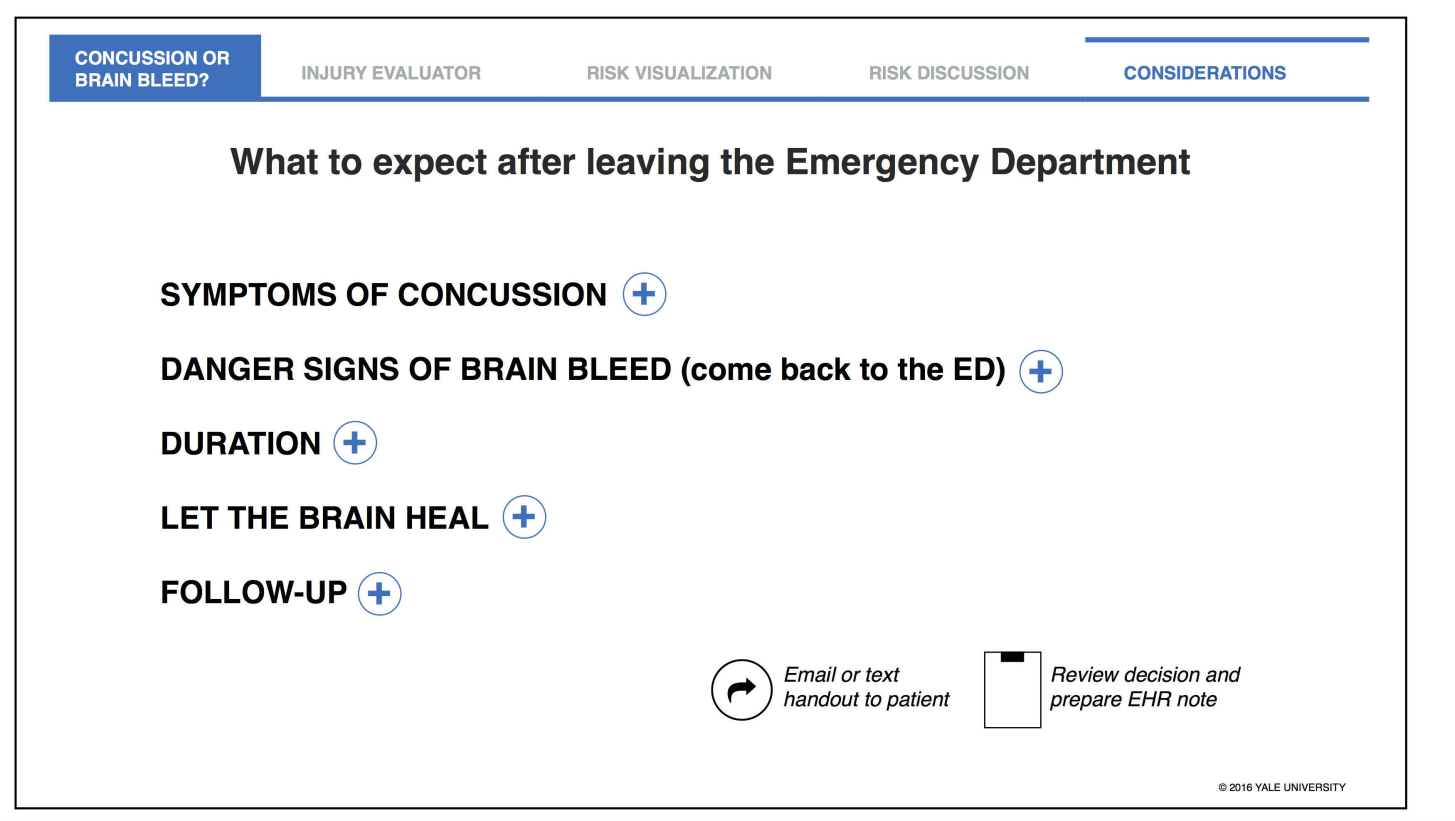
Considerations screen for low-risk patients after beta testing.

Beta testing was conducted over 6 weeks with 4 attending emergency physicians in the care of 7 low-risk, minor head injury ED patients. The final Considerations section for low-risk patients was revised based on user feedback that it was too busy. Prior to beta testing, this section had a wall of text including a large inventory of sections that could be discussed at the clinician’s discretion. Beta testing revealed just a checklist with the option to expand sufficed. The section’s content remained relatively unchanged with the format converted to a checklist with single-tap dropdown options that provided more information (via hypertext) when specifically selected (Figure 8). Readability increased with limited distractions while remaining flexible to differing clinician practice styles and individual patient needs.

We developed a work-around for integration with EHR workflow using Epic (Epic Systems Corp) SmartPhrases (Multimedia Appendix 4). This charting tool allows clinicians to autopopulate text using shorthand. SmartPhrases allow rapid documentation of use of the *Concussion or Brain Bleed* app in the EHR.

## Discussion

### Principal Findings

A total of 16 successive iterations have resulted in a tool that integrates Canadian CT Head Rule CDS at point of care with a patient decision aid to promote conversation around individualized risk and patients’ specific concerns. Design, development, and formative evaluation were informed by the philosophy that technology can accelerate the provision of evidence-based care that is efficient and empathic, effectively reducing unnecessary care [17,19,24]. The user can traverse the app in its entirety in 3 to 5 screen taps. *Concussion or Brain Bleed* addresses the human factors that research demonstrates are critical for optimizing CT use in minor head injury by creating the time and space for conversation between patients and their clinician [16]. The app equips clinicians to foster trust and manage patient expectations in a fast-paced ED environment characterized by uncertainty and high emotions.

We elected a formative evaluation inclusive of usability evaluation—namely, the well-established practice-based, patient-centered, and participatory decision aid development process adapted for our computerized tool [18,19,41-44]. It allowed for problematic elements of the prototype to be rapidly identified and addressed and usability of unchanged elements to be tested by multiple users through existing and subsequent iterations. The final product is refined based on user input and represents the culmination of rigorous testing in simulations and real-world clinical encounters. Feedback from a multidisciplinary team has been incorporated with the express goal of practicality and usability. Our tool addresses multiple items across the dimensions of the International Patient Decision Aids Standards including the use of a systematic development process, presenting information on probabilities of outcomes and using the scientific literature on which content is based, conveyed with plain language [43]. Involving end-users and a variety of clinicians in both simulated and real-world clinical environments in an iterative process ensures that the format and information content of our tool is responsive to user preferences and the complexities of decision context.

### Comparison With Prior Work

CDS is most effective when it is part of the clinician’s workflow at the time and location of decision making [27,28]. Decision support strategies to date have focused on physician behavior [25-27]. By bringing CDS to the point of care and integrating it with a decision aid on a tablet computer shared by the patient and clinician, *Concussion or Brain Bleed* could improve both the quantity and quality of time at the beside. Patient decision aids have been established as an effective way to translate evidence-based care into practice [29,50]. Visualizing benefits and harms can lead to increased patient knowledge and involvement in decision making, greater satisfaction with the decision-making process, and optimal health outcomes consistent with patient values and preferences [29]. Patient involvement begins with the development of a partnership and includes participation in information exchange, deliberation, and decision making [51]. Patients—even older patients with more experience in a historically paternalistic mode—report great interest in getting involved in similar decision making again [52,53]. While paper-based patient decision aids are beneficial, they do not provide CDS for the clinician and are static in nature with regard to patient-specific estimates and concerns [18,29,44,50]. Tablet computers retain the portability and usability of a paper-based decision aid while also providing the customization and flexibility of computerized CDS with regard to patient-specific risk visualization. In developing a digital tool, we also have the additional benefit of having a database to collect, edit, store, and retrieve data generated by the tool and further reduce workflow burden through direct integration with EHR systems.

### Conclusions

The fight to stem medical overuse will require the use of disruptive technologies—often innovative but simple, high-value solutions that can be widely adopted and easily used. In creating this patient-centered clinical decision support tool, we aim to decrease CT use for minor head injury. This tool combines evidence-based practices with patient engagement that establishes trust, empowers active participation, and addresses patient concerns and uncertainty about their condition at the point of care. It helps clinicians to determine who needs a CT and then helps patients to understand why. We hypothesize that, when implemented, the *Concussion or Brain Bleed* app will support—not hinder—the clinician-patient relationship, safely reduce CT use, and improve the patient experience of care.

## Appendix

Multimedia Appendix 1 Usability evaluation standardized cases.

Multimedia Appendix 2 Usability evaluation postuse semistructured interview guide.

Multimedia Appendix 3 Head computed tomography decision tool for the emergency department prototype research questions.

Multimedia Appendix 4 Beta testing instructions for using the app and Epic SmartPhrases to document use of the tool in the electronic health record.

## Abbreviations

CDS: clinical decision support
CT: computed tomography
ED: emergency department
EHR: electronic health record
IRB: institutional review board
UI: user interface
UX: user experience

## References

[1] Dematteo CA, Hanna SE, Mahoney WJ, Hollenberg RD, Scott LA, Law MC, Newman A, Lin CA, Xu L (2010). “My child doesn't have a brain injury, he only has a concussion”. Pediatrics.

[2] Stiell IG, Wells GA, Vandemheen K, Clement C, Lesiuk H, Laupacis A, McKnight RD, Verbeek R, Brison R, Cass D, Eisenhauer ME, Greenberg G, Worthington J (2001). The Canadian CT Head Rule for patients with minor head injury. Lancet.

[3] Stiell IG, Clement CM, Rowe BH, Schull MJ, Brison R, Cass D, Eisenhauer MA, McKnight RD, Bandiera G, Holroyd B, Lee JS, Dreyer J, Worthington JR, Reardon M, Greenberg G, Lesiuk H, MacPhail I, Wells GA (2005). Comparison of the Canadian CT Head Rule and the New Orleans Criteria in patients with minor head injury. JAMA.

[4] Stiell IG, Clement CM, Grimshaw JM, Brison RJ, Rowe BH, Lee JS, Shah A, Brehaut J, Holroyd BR, Schull MJ, McKnight RD, Eisenhauer MA, Dreyer J, Letovsky E, Rutledge T, Macphail I, Ross S, Perry JJ, Ip U, Lesiuk H, Bennett C, Wells GA (2010). A prospective cluster-randomized trial to implement the Canadian CT Head Rule in emergency departments. CMAJ.

[5] Smits M, Dippel DW, de Haan GG, Dekker HM, Vos PE, Kool DR, Nederkoorn PJ, Hofman PA, Twijnstra A, Tanghe HL, Hunink MG (2005). External validation of the Canadian CT Head Rule and the New Orleans Criteria for CT scanning in patients with minor head injury. JAMA.

[6] Jagoda AS, Bazarian JJ, Bruns JJ, Cantrill SV, Gean AD, Howard PK, Ghajar J, Riggio S, Wright DW, Wears RL, Bakshy A, Burgess P, Wald MM, Whitson RR, American College of Emergency Physicians, Centers for Disease ControlPrevention (2008). Clinical policy: neuroimaging and decisionmaking in adult mild traumatic brain injury in the acute setting. Ann Emerg Med.

[7] Papa L, Stiell IG, Clement CM, Pawlowicz A, Wolfram A, Braga C, Draviam S, Wells GA (2012). Performance of the Canadian CT Head Rule and the New Orleans Criteria for predicting any traumatic intracranial injury on computed tomography in a United States Level I trauma center. Acad Emerg Med.

[8] Melnick ER, Szlezak CM, Bentley SK, Dziura JD, Kotlyar S, Post LA (2012). CT overuse for mild traumatic brain injury. Jt Comm J Qual Patient Saf.

[9] Parma C, Carney D, Grim R, Bell T, Shoff K, Ahuja V (2014). Unnecessary head computed tomography scans: a level 1 trauma teaching experience. Am Surg.

[10] Korley FK, Morton MJ, Hill PM, Mundangepfupfu T, Zhou T, Mohareb AM, Rothman RE (2013). Agreement between routine emergency department care and clinical decision support recommended care in patients evaluated for mild traumatic brain injury. Acad Emerg Med.

[11] Sharp AL, Nagaraj G, Rippberger EJ, Shen E, Swap CJ, Silver MA, McCormick T, Vinson DR, Hoffman JR (2017). Computed tomography use for adults with head injury: describing likely avoidable emergency department imaging based on the Canadian CT Head Rule. Acad Emerg Med.

[12] Melnick ER (2017). Big versus small data and the generalizability of the rate of computed tomography overuse in minor head injury. Acad Emerg Med.

[13] American College of Emergency Physicians (2014). Ten Things Physicians and Patients Should Question.

[14] Ip IK, Raja AS, Gupta A, Andruchow J, Sodickson A, Khorasani R (2015). Impact of clinical decision support on head computed tomography use in patients with mild traumatic brain injury in the ED. Am J Emerg Med.

[15] Sharp A, Huang B, Tang T, Shen E, Melnick E, Venkatesh A (2017). Implementation of the Canadian CT Head Rule and its effect on utilization of computed tomography among patients with head injury. Ann Emerg Med (in press).

[16] Melnick ER, Shafer K, Rodulfo N, Shi J, Hess EP, Wears RL, Qureshi RA, Post LA (2015). Understanding overuse of computed tomography for minor head injury in the emergency department: a triangulated qualitative study. Acad Emerg Med.

[17] Melnick ER (2015). How to make less more: empathy can fill the gap left by reducing unnecessary care. BMJ.

[18] Montori VM, Breslin M, Maleska M, Weymiller AJ (2007). Creating a conversation: insights from the development of a decision aid. PLoS Med.

[19] Melnick ER, Lopez K, Hess EP, Abujarad F, Brandt CA, Shiffman RN, Post LA (2015). Back to the bedside: developing a bedside aid for concussion and brain injury decisions in the emergency department. EGEMS (Wash DC).

[20] Toll E (2012). A piece of my mind. The cost of technology. JAMA.

[21] Ratanawongsa N, Barton JL, Lyles CR, Wu M, Yelin EH, Martinez D, Schillinger D (2016). Association between clinician computer use and communication with patients in safety-net clinics. JAMA Intern Med.

[22] Sinsky Christine, Tutty Michael, Colligan Lacey (2017). Allocation of Physician Time in Ambulatory Practice. Ann Intern Med.

[23] Hill RG, Sears LM, Melanson SW (2013). 4000 clicks: a productivity analysis of electronic medical records in a community hospital ED. Am J Emerg Med.

[24] Gellert G, Webster S, Gillean J, Melnick E, Kanzaria H (2017). Should US doctors embrace electronic health records?. BMJ.

[25] Hunt DL, Haynes RB, Hanna SE, Smith K (1998). Effects of computer-based clinical decision support systems on physician performance and patient outcomes: a systematic review. JAMA.

[26] Garg AX, Adhikari NK, McDonald H, Rosas-Arellano MP, Devereaux PJ, Beyene J, Sam J, Haynes RB (2005). Effects of computerized clinical decision support systems on practitioner performance and patient outcomes: a systematic review. JAMA.

[27] Kawamoto K, Houlihan CA, Balas EA, Lobach DF (2005). Improving clinical practice using clinical decision support systems: a systematic review of trials to identify features critical to success. BMJ.

[28] Karsh B (2009). Clinical practice improvement and redesign: how change in workflow can be supported by clinical decision support.

[29] Stacey D, Légaré F, Col NF, Bennett CL, Barry MJ, Eden KB, Holmes-Rovner M, Llewellyn-Thomas H, Lyddiatt A, Thomson R, Trevena L, Wu JH (2014). Decision aids for people facing health treatment or screening decisions. Cochrane Database Syst Rev.

[30] Rosenbaum L (2015). Transitional chaos or enduring harm? The EHR and the disruption of medicine. N Engl J Med.

[31] Barnett ML, Mehrotra A, Jena AB (2016). Adverse inpatient outcomes during the transition to a new electronic health record system: observational study. BMJ.

[32] Concussion or Brain Bleed?.

[33] Dong T, Churchill E, Nichols J (2016). Understanding the challenges of designing and developing multi-device experiences.

[34] Panhale M (2016). Introduction to Mobile Application Development Ecosystems: Beginning Hybrid Mobile Application Development.

[35] Chung K, Kim J, Park RC (2015). Knowledge-based health service considering user convenience using hybrid Wi-Fi P2P. Inf Technol Manag.

[36] Goldberg L, Lide B, Lowry S, Massett HA, O'Connell T, Preece J, Quesenbery W, Shneiderman B (2011). Usability and accessibility in consumer health informatics current trends and future challenges. Am J Prev Med.

[37] Curry LA, Nembhard IM, Bradley EH (2009). Qualitative and mixed methods provide unique contributions to outcomes research. Circulation.

[38] Fonteyn ME, Kuipers B, Grobe SJ (1993). A description of think aloud method and protocol analysis. Qual Health Res.

[39] Floch M, Walker W, Ringel Y (1998). An ecological approach to interface design.

[40] Vicente K (2002). Ecological interface design: progress and challenges. Hum Factors.

[41] Hess EP, Wyatt KD, Kharbanda AB, Louie JP, Dayan PS, Tzimenatos L, Wootton-Gorges SL, Homme JL, Pencille RN, LeBlanc A, Westphal JJ, Shepel K, Shah ND, Branda M, Herrin J, Montori VM, Kuppermann N (2014). Effectiveness of the head CT choice decision aid in parents of children with minor head trauma: study protocol for a multicenter randomized trial. Trials.

[42] Melnick ER, Probst MA, Schoenfeld E, Collins SP, Breslin M, Walsh C, Kuppermann N, Dunn P, Abella BS, Boatright D, Hess EP (2016). Development and testing of shared decision making interventions for use in emergency care: a research agenda. Acad Emerg Med.

[43] Elwyn G, O'Connor AM, Bennett C, Newcombe RG, Politi M, Durand M, Drake E, Joseph-Williams N, Khangura S, Saarimaki A, Sivell S, Stiel M, Bernstein SJ, Col N, Coulter A, Eden K, Härter M, Rovner MH, Moumjid N, Stacey D, Thomson R, Whelan T, van der Weijden T, Edwards A (2009). Assessing the quality of decision support technologies using the International Patient Decision Aid Standards instrument (IPDASi). PLoS One.

[44] Coylewright M, Shepel K, Leblanc A, Pencille L, Hess E, Shah N, Montori VM, Ting HH (2012). Shared decision making in patients with stable coronary artery disease: PCI choice. PLoS One.

[45] Breslin M, Mullan RJ, Montori VM (2008). The design of a decision aid about diabetes medications for use during the consultation with patients with type 2 diabetes. Patient Educ Couns.

[46] Hess EP, Knoedler MA, Shah ND, Kline JA, Breslin M, Branda ME, Pencille LJ, Asplin BR, Nestler DM, Sadosty AT, Stiell IG, Ting HH, Montori VM (2012). The chest pain choice decision aid: a randomized trial. Circ Cardiovasc Qual Outcomes.

[47] Melnick E, Szlezak C, Dziura J, Stiell I (2013). Risk estimates for the Canadian CT Head Rule in patients with minor head injury.

[48] Kurz-Milcke E, Gigerenzer G, Martignon L (2008). Transparency in risk communication: graphical and analog tools. Ann NY Acad Sci.

[49] Kent DM, Shah ND (2011). Personalizing evidence-based primary prevention with aspirin: individualized risks and patient preference. Circ Cardiovasc Qual Outcomes.

[50] Hess EP, Hollander JE, Schaffer JT, Kline JA, Torres CA, Diercks DB, Jones R, Owen KP, Meisel ZF, Demers M, Leblanc A, Shah ND, Inselman J, Herrin J, Castaneda-Guarderas A, Montori VM (2016). Shared decision making in patients with low risk chest pain: prospective randomized pragmatic trial. BMJ.

[51] Charles C, Gafni A, Whelan T (1997). Shared decision-making in the medical encounter: what does it mean? (or it takes at least two to tango). Soc Sci Med.

[52] Mullan RJ, Montori VM, Shah ND, Christianson TJ, Bryant SC, Guyatt GH, Perestelo-Perez LI, Stroebel RJ, Yawn BP, Yapuncich V, Breslin MA, Pencille L, Smith SA (2009). The diabetes mellitus medication choice decision aid: a randomized trial. Arch Intern Med.

[53] Weymiller AJ, Montori VM, Jones LA, Gafni A, Guyatt GH, Bryant SC, Christianson TJ, Mullan RJ, Smith SA (2007). Helping patients with type 2 diabetes mellitus make treatment decisions: statin choice randomized trial. Arch Intern Med.

