# Supplementary material for: Patient-Centered Decision Support: Formative Usability Evaluation of Integrated Clinical Decision Support With a Patient Decision Aid for Minor Head Injury in the Emergency Department
Source: J Med Internet Res. 2017 May 19;19(5):e174. doi: 10.2196/jmir.7846 (PMC5457532; doi:10.2196/jmir.7846)
Supplement: Multimedia Appendix 1 [file jmir_v19i5e174_app1.pdf]

Multimedia Appendix 1. Usability evaluation standardized cases

Connie A. Case, MRN 234567

A 32-year old female presents to the emergency department following a closed head injury. She notes that she asked her husband to pass her the cordless phone, but he threw it to her and it struck her in the head. The phone struck her in the right pareitotemporal area where she now has some swelling. There are no signs of basilar skull fracture, open or depressed skull fracture on exam.
The patient complains of a headache since this injury occurred two hours ago and that she vomited once and thinks she may have “stomach flu.” But when she looked up these symptoms on WebMD, it advised she go directly to the emergency department “to get checked out.” She denies loss of consciousness, amnesia before or after the injury, confusion, seizure, focal weakness or numbness. She is healthy and denies any past medical history or prescription medication use. She asks you, “Do I need a CAT scan?”

Vital Signs:
Temperature 98.0 F
Pulse 68
Blood Pressure 115/77
Respiratory Rate 14
GCS 15

<u>Additional instructions for standardized patient:</u>
Despite her headache and vomiting, this patient is actually very low risk because she does not make criteria for minor head injury, i.e., no loss of consciousness, amnesia, or altered mentation. The presence of the headache and vomiting are, however, concerning for a concussion.

The patient does not “expect” a CT scan and will be satisfied with her evaluation and without a CT scan as long as the physician takes the time to:

1. Listen and care for the patient as a person
2. Identify and address the patient’s concerns including: concussion, her symptoms, and why a CT will not help
3. And does not dwell on radiation risk to scare the patient

If the physician does not engage the patient on these points, then the patient will push harder for a CT, e.g., bring up Natasha Richardson or a family member or primary doctor who says “you shouldn’t come home till you get that scan”

Edith B. Case, MRN 876543

A 94-year old female is brought in by ambulance for evaluation from a nursing home where per report she fell while trying to get out of bed unassisted. The patient is awake, alert, and oriented x3. She recalls the event and states, “I feel fine, doc. Can I just go home and get back to bed?” You note some mild, soft tissue swelling over her right occiput on exam without evidence of open or depressed skull fracture. She denies nausea, vomiting, headache, loss of consciousness, amnesia before or after the injury, confusion, seizure, focal weakness or numbness. The patient appears spry and notes a past medical history significant for hypertension. She states that she takes lisinopril, “baby” aspirin, and “vitamins.”

Vital Signs:
Temperature 98.4 F
Pulse 72
Blood Pressure 128/83
Respiratory Rate 16
GCS 15

<u>Additional instructions for standardized patient:</u>
Despite her advanced age, this patient is actually very low risk because she does not make criteria for minor head injury, i.e., no loss of consciousness, amnesia, or altered mentation.

The patient does not want a CT scan because the results will not change the fact that she’s lived a long, happy, healthy life. Even if a clinically important brain injury is found on scan, the patient states she would not want to be hospitalized or undergo surgery. The patient feels very strongly about this and would, in fact, rather not know if she has a life-threatening condition—preferring instead to continue her daily routine and die in her sleep. The patient will not volunteer these preferences unless the physician prompts the patient to provide it. If the physician does not engage the patient, she will not argue about getting a CT.

Henry C. Case, MRN 345678

A 47-year old male is brought in by ambulance to the emergency department following a closed head injury. The patient does not recall the event or if he lost consciousness. He remembers that he was doing some home repairs, and his wife told him that she heard a loud thud and found him at the bottom of their basement staircase. He does not remember any events in the 30 minutes prior to the injury, so he cannot report how many steps he fell down. There are no signs of basilar skull fracture, open or depressed skull fracture on exam. The patient complains of a headache and two episodes of vomiting since the injury. He denies confusion, seizure, focal weakness or numbness. He is healthy apart from a history of hypertension for which he takes hydrochlorthiazide.

Vital Signs:
Temperature 99.0 F
Pulse 81
Blood Pressure 152/86
Respiratory Rate 20
GCS 15

<u>Additional instructions for standardized patient:</u>
This case is intended to serve as a “control” for a case when a CT should be ordered and the patient is agreeable to the scan. The patient is high risk for need for neurosurgical intervention due to the fact that he vomited twice. Furthermore, his amnesia of >30 minutes and likely fall from >5 stairs also make him moderate risk for clinically important brain injury. Neurologic exam should still be non-focal. The patient should be lying flat in the stretcher to simulate screen sharing in a supine position and to cue the physician user that this patient is “sicker.”

Norbert D. Case, MRN 765432

A 47-year old male is brought in by ambulance to the emergency department following a closed head injury. The patient does not recall the event or if he lost consciousness. He remembers that he was doing some home repairs, and his wife told him that she heard a loud thud and found him at the bottom of their basement staircase. He does not remember any events in the 30 minutes prior to the injury, so he cannot report how many steps he fell down. There are no signs of basilar skull fracture, open or depressed skull fracture on exam. The patient complains of a headache and two episodes of vomiting since the injury. He denies confusion, seizure, focal weakness or numbness. He is healthy apart from a history of hypertension for which he takes hydrochlorthiazide.

Vital Signs:
Temperature 99.0 F
Pulse 81
Blood Pressure 152/86
Respiratory Rate 20
GCS 15

Additional instructions for standardized patient:
This case is intended to serve as a "control" for a case when a CT should be ordered and the patient is **refuses** the scan. The patient is high risk for need for neurosurgical intervention due to the fact that he vomited twice. Furthermore, his amnesia of >30 minutes and likely fall from >5 stairs also make him moderate risk for clinically important brain injury. Neurologic exam should still be non-focal. The patient should be lying flat in the stretcher to simulate screen sharing in a supine position and to cue the physician user that this patient is "sicker."

The patient does not want a CT scan due to concerns for the risk of cancer due to radiation from the scan. The patient will change his mind and agree to the scan as long as the physician takes the time to:

1. Listen and care for the patient as a person
2. Identify and address the patient's concerns radiation risk

If the physician does not engage the patient on these points, then the patient will push harder to leave the ED without the scan.
