# Supplementary material for: Patient-Centered Decision Support: Formative Usability Evaluation of Integrated Clinical Decision Support With a Patient Decision Aid for Minor Head Injury in the Emergency Department
Source: J Med Internet Res. 2017 May 19;19(5):e174. doi: 10.2196/jmir.7846 (PMC5457532; doi:10.2196/jmir.7846)
Supplement: Multimedia Appendix 2 [file jmir_v19i5e174_app2.pdf]

Multimedia Appendix 2. Usability evaluation post-use semi-structured interview guide

A structured interview will be conducted to gather information about overall strategies, cues, and other decision processes they used throughout the session and how having the decision support prototype impacted the decision-making process.

Decision Point Identification:

Will you give me a quick run through of the case?

Listen for: Decision points, shifts in situation assessment, places to probe, gaps in the story, gaps in the timeline, conceptual leaps, anomalies/violated expectations, errors, ambiguous cues, individual differences

Flags: I just knew… It felt right… I guessed… It was just a gut feeling… Something felt wrong… I've seen it before… It depends…

Deepening: The story behind the story; ask questions until you understand the incident, repeat back confusing points

- If you were signing out this case at this point, what would you be sure to tell the relief person?

Listen for: Critical decisions, cues and their implications, ambiguous cues, strategies, anomalies/violated expectations

1. **Cues:** What was it about the situation that let you know what to do?

2. **Deepening:** What were your overriding concerns during your conversation?

3. **Challenges:** Why is this conversation so difficult to do?

4. **Typicality:** Is this what you would usually do?

5. **Shared Decision Making:** What contribution did the patient's expectations, concerns and values play in what you did?

6. **Goals:** What were you trying to do or accomplish?

7. **Constraints:** What kept you from doing what you would've liked to do?

8. **Options:** What did you want to do, but could not?

9. **Information needs:** What could have helped you there?

10. **Time Pressure/Big picture:** What was driving the pace of the conversation?

11. **Strategy/Rule of Thumb:** What worked well about this conversation with this patient?

12. **Rationale/Information Needs:** Were you following a particular rule or approach?

13. **Information needs:** What information did you use in making this decision?

What if Queries (expert-novice differences) use "what if" questions to tease out specific elements

Listen for: other possible courses of action, other potential interpretations, expert-novice differences, potential errors

1. **Errors:** Where might someone without your experience have made a mistake?

2. **Strategy/Rule of thumb:**
   - How would you have made the same decision at an earlier point in your career?

   or
   - Why would this decision have turned out differently if you or someone with your level/skill had not been caring for this patient?

3. **Closing:** Is there anything important to know about making this decision that I should have asked but didn't? Did I forget anything?

After the participant has completed the session, a usability feedback questionnaire (using the user- centered design approach) will be administered to obtain satisfaction ratings about the tool's ease-of-use, usefulness, and perceived value, perceived time of SDM conversation, explain why final decision made the way it was, open-ended user

comments
