# Supplementary material for: Patient-Centered Decision Support: Formative Usability Evaluation of Integrated Clinical Decision Support With a Patient Decision Aid for Minor Head Injury in the Emergency Department
Source: J Med Internet Res. 2017 May 19;19(5):e174. doi: 10.2196/jmir.7846 (PMC5457532; doi:10.2196/jmir.7846)
Supplement: Multimedia Appendix 3 [file jmir_v19i5e174_app3.pdf]

Multimedia Appendix 3. Head CT Decision Tool for the Emergency Department Prototype Research Questions

EMERGENCY DEPARTMENT CONTEXT QUESTIONS

Observations in the ED with head trauma patients and other analogous patients who may be making a similar decision about whether or not to get a CT scan.

Initial research will attempt to capture patients in the ED in real time. Research sessions might involve 90-180 minute sessions in the ED.

Scenario

Ask patient and physician to review the tool as if they were discussing head trauma - substituting their own concerns for the ones in the tool where possible.

Observations

How do the physician and patient position themselves when using the tool?
How does the physician interact with the tool?
How does the physician share the tool with the patient?
How does the patient interact with the tool?
What questions does the patient ask?
How does the physician answer questions?
Do family members or people accompanying the patient get involved with the tool?

to Patient:

Did the concerns/issues seem relevant?

Do you think you would have talked about these issues without the tool?

Are there concerns you might add?

Where the responses to your questions/concerns satisfactory?

What was your impression of the risk? (if the patient didn’t view this on their own, show them now.)

Do you feel that the tool made it easier or harder to talk with the doctor?

to Physician:

What was the experience of using the tool like? What might you do differently if you were to use it again?

How confident did you feel answering any questions that came up in the conversation?

Do you feel that the tool made it easier or harder to talk with the patient?

HEAD TRAUMA CONTENT QUESTIONS

People who came to the ED with head trauma in the past and made this decision or would have been in a position to use this tool.

Initial research will likely be during 60 minute interviews with former patients known to the study group who agree to come in and participate.

<u>Scenario</u>

(1) Ask patient to describe their actual visit to the ED for head trauma. Do they remember what their concerns were? Were options shared with them? What did they decide to do? Why?

(2) With a physician playing the part of the physician, try to reenact the ED visit now using the tool.

<u>to Patient:</u>

Did your actual ED visit involve shared decision making?

What topics did you talk about with your ED doctor?

Do you remember what concerns were on your mind?

Were these questions/concerns that you had?

Do you think you would have talked about these issues without the tool?

Are there concerns you might add?

Where the responses to your questions/concerns satisfactory?

if you were to make the decision now, after using this tool, do you think you'd make the same decision?
