# Supplementary material for: Patient-Centered Decision Support: Formative Usability Evaluation of Integrated Clinical Decision Support With a Patient Decision Aid for Minor Head Injury in the Emergency Department
Source: J Med Internet Res. 2017 May 19;19(5):e174. doi: 10.2196/jmir.7846 (PMC5457532; doi:10.2196/jmir.7846)
Supplement: Multimedia Appendix 4 [file jmir_v19i5e174_app4.pdf]

Multimedia Appendix 4. Beta-testing instructions for using the app and Epic smart phrases to document use of tool in EHR.

Instructions

- Once you are in the app, it should be relatively self-explanatory. The purpose of what you are doing now is to test the software—fix bugs and tweak things that don't make sense. So, please give me feedback (by follow-up survey or email) with any questions or issues that arise.

- The app is intended to be used to **discuss the need for CT** (e.g., after H&P are done) for patients with minor head injury that are not on prescription anticoagulants, don't have a bleeding disorder, and did not seize. It should take **3-7 minutes** to go through it. The goal is to engage patients and safely reduce CT use in low risk patients. So, for low risk patients this investment of time will save you and the patient time on the back end of not having to order and follow-up a negative study.

- The purpose of the app is to help you **risk stratify** the patient using the Canadian CT Head Rule. Then to share the **risk visualization** with the patient (optional) as well as serve as a platform for **discussion of risk and considerations** of what to expect if you do or don't get a CT.

    - Remember that the original Canadian CT Head Rule studies included patients who had LOC, amnesia, or altered sensorium as well as GCS 13-15, so these risk visualizations are actually an over-estimate for most of our head injury patients in the ED.

Epic smart phrases

- **.CCHRLOWNOCT** I have used the "Concussion or Brain Bleed?" decision aid to discuss the decision about getting a CT scan with this patient. This patient's injury is **low** risk based on the Canadian CT Head Rule. ACEP and Choosing Wisely recommend avoiding CT of the head in emergency department patients with minor head injury who are at low risk based on validated decision rules such as the Canadian CT Head Rule. We discussed the patient's risks of: (1) need for neurosurgical intervention to be 0.0%, (2) clinically important brain injury to be 1.1%, (3) any brain injury by CT to be 2.7%. After discussing and considering the patient's unique circumstances and the pros and cons of the alternatives, we decided the patient should go home now without a CT.

- **.CCHRLOWYESCT** I have used the "Concussion or Brain Bleed?" decision aid to discuss the decision about getting a CT scan with this patient. This patient's injury is **low** risk based on the Canadian CT Head Rule. We discussed the patient's risks of: (1) need for neurosurgical intervention to be 0.0%, (2) clinically important brain injury to be 1.1%, (3) any brain injury by CT to be 2.7%. After discussing and considering the patient's unique circumstances and the pros and cons of the alternatives, we have decided to obtain a CT scan.

- **.CCHRMEDNOCT** I have used the "Concussion or Brain Bleed?" decision aid to discuss the decision about getting a CT scan with this patient. This patient's injury is **medium** risk based on the Canadian CT Head Rule. AWe discussed the patient's risks of: (1) need for neurosurgical intervention to be 0.0%, (2) clinically important brain injury to be 3.8%, (3) any brain injury by CT to be 6.9%. After discussing and considering the patient's unique circumstances and the pros and cons of the alternatives, we decided the patient should go home now without a CT.

- **.CCHRMEDNOCT** I have used the "Concussion or Brain Bleed?" decision aid to discuss the decision about getting a CT scan with this patient. This patient's injury is **medium** risk based on the Canadian CT Head Rule. The Canadian CT Head Rule recommends obtaining a CT in medium risk patients. We discussed the patient's risks of: (1) need for neurosurgical intervention to be 0.0%, (2) clinically important brain injury to be 3.8%, (3) any brain injury by CT to be 6.9%. After discussing and considering the patient's unique circumstances and the pros and cons of the alternatives, we have decided to obtain a CT scan.

- **.CCHRHINOCT** I have used the "Concussion or Brain Bleed?" decision aid to discuss the decision about getting a CT scan with this patient. This patient's injury is **high** risk based on the Canadian CT Head Rule. We discussed the patient's risks of: (1) need for neurosurgical intervention to be 4.5%, (2) clinically important brain injury to be 21.6%, (3) any brain injury by CT to be 26.7%. After discussing and considering the patient's unique circumstances and the pros and cons of the alternatives, we decided the patient should go home now without a CT.

- **.CCHRHIYESCT** I have used the "Concussion or Brain Bleed?" decision aid to discuss the decision about getting a CT scan with this patient. This patient's injury is **high** risk based on the Canadian CT Head Rule. The Canadian CT Head Rule recommends obtaining a CT in high risk patients. We discussed the patient's risks of: (1) need for neurosurgical intervention to be 4.5%, (2) clinically important brain injury to be 21.6%, (3) any brain injury by CT to be 26.7%. After discussing and considering the patient's unique circumstances and the pros and cons of the alternatives, we have decided to obtain a CT scan.
